# The Effectiveness of Orthopaedic Insoles in Reducing Plantar Pressure and Preventing the Onset of First-Time or the Recurrence of Foot Ulcers in Diabetic Patients—A Systematic Review

**DOI:** 10.3390/jcm15218740

**Published:** 2026-11-09

**Authors:** Marta Izquierdo-Renau, Esther García-Morales, Iván Julián-Rochina, Raquel Alabau-Dasi, Enrique Sanchis-Sales, José Luis Lázaro-Martínez, Aroa Tardáguila-García

**Affiliations:** 1 Nursing Department, University of Valencia, 46010 Valencia, Spain; marta.izquierdo-renau@uv.es (M.I.-R.); ivan.julian@uv.es (I.J.-R.); raquel.alabau@uv.es (R.A.-D.);; 2 Prevention and Treatment of Diabetic Foot Syndrome Research Group, University of Valencia, 46010 Valencia, Spain; 3 Diabetic Foot Unit, Clínica Universitaria de Podología, Facultad de Enfermería, Fisioterapia y Podología, Instituto de Investigación Sanitaria del Hospital Clínico San Carlos (IdISSC), Universidad Complutense de Madrid, 28040 Madrid, Spainaroa.tardaguila@ucm.es (A.T.-G.); 4 Frailty Research Organized Group (FROG), University of Valencia, 46010 Valencia, Spain

**Keywords:** insoles, foot support, injury, diabetes-related foot disease, diabetic foot

## Abstract

**Background/Objectives**: Prevention of the first diabetic foot ulcer and subsequent ulcer recurrence is a key priority in diabetes care. Foot orthoses are used to redistribute plantar pressure and protect high-risk areas; however, their effectiveness in preventing ulceration remains uncertain. This systematic review aimed to evaluate the effectiveness of foot orthoses in modifying plantar pressure and preventing first or recurrent diabetic foot ulcers. **Methods**: A systematic review was conducted following the Preferred Reporting Items for Systematic Reviews and Meta-Analyses (PRISMA) methodology. PubMed, Web of Science, and Embase were searched. Studies were selected according to predefined eligibility criteria related to publication type, language, patient characteristics, and the use of foot orthoses. Methodological quality and risk of bias were assessed using Joanna Briggs Institute critical appraisal tools. **Results**: The search identified 963 records. After screening and full-text assessment, 12 articles met the inclusion criteria; one additional article was identified and included subsequently, resulting in 13 studies. Overall, foot orthoses reduced plantar pressure, particularly beneath the metatarsal heads. However, the available evidence directly assessing the prevention of first or recurrent diabetic foot ulceration was limited. **Conclusions**: Foot orthoses may contribute to diabetic foot ulcer prevention by reducing plantar pressure in high-risk areas. Nevertheless, further well-designed longitudinal studies are needed to determine whether these biomechanical improvements translate into a reduction in ulcer incidence and recurrence.

## 1. Introduction

The development of a diabetic foot ulcer (DFU) can lead to lower limb amputation, with risk increasing when diabetic neuropathy coexists with peripheral vascular disease [1]. The 5-year mortality rate following diabetic foot amputation also exceeds that of several cancers, including prostate and breast cancer [2]. This condition produces substantial direct costs related to healthcare, hospitalisation and surgery, as well as indirect costs, because patients with lesions experience reduced productivity and diminished quality of life [3]. Prevention strategies and multidisciplinary management are essential to limit expenditure and major complications [4]. Through comprehensive foot care, therapeutic footwear, pressure control and temperature monitoring, the incidence of DFUs could be reduced by 75% [5,6].

Risk factors for the development and recurrence of DFUs include male sex, smoking history, living alone, history of plantar ulcer, diabetic retinopathy, diabetic nephropathy, diabetic peripheral neuropathy, foot deformity and peripheral arterial disease [4]. Although DFUs have multifactorial aetiology, deformity from motor neuropathy exposes specific regions of the foot to elevated plantar pressure, notably the metatarsal heads and toes, increasing ulcer susceptibility [7,8,9]. Lobmann et al. [10] demonstrated an association between neuropathy severity and increased pressures.

Non-enzymatic glycosylation of structural proteins such as collagen reduces elasticity in joint capsules, ligaments and tendons, limiting joint range of motion, particularly at the ankle, and promoting higher plantar pressure [11,12]. These pressures damage blood vessels, limit nutrient supply and produce tissue ischaemia, culminating in ulceration [13]. When assessing pressure between foot and footwear, a maximum threshold of 200 kPa is considered indicative of increased ulceration risk, although some patients ulcerate below this threshold [14,15]. At the foot–ground interface, pressures >700 kPa show high specificity and sensitivity in identifying individuals who are at greatest risk [16], although Veves et al. [17] reported even higher predictive values (1026 kPa). Given the central role of plantar pressure in ulcer pathogenesis, its assessment is essential, particularly in patients with diabetes and previous lesions, with emphasis on reducing both pressure magnitude and exposure time [18,19].

Therapeutic footwear and foot orthoses are used to prevent ulceration or re-ulceration by reducing high pressures on vulnerable areas [20]. To be effective, these devices must achieve either a 30% reduction in plantar pressure relative to barefoot or footwear-only conditions or a maximum pressure <200 kPa [21]. Ulbrecht et al. [22] demonstrated that the use of custom-made orthoses (tailored to the foot’s morphology and guided by its pressure profiles) significantly reduces the recurrence of ulcers in the metatarsal region, showing greater efficacy than standard devices. Elgohary et al. [23] similarly found that foot orthoses reduce maximum plantar pressure and the pressure–time integral in patients with ulcers, indicating substantial benefit for preventing re-ulceration.

The aim of this review is to assess the effectiveness of foot orthoses in modifying plantar pressure and preventing first or recurrent ulceration. Secondarily, we examine the types of orthoses (customised or non-customised), commonly used materials and footwear employed in combination with orthoses for ulcer prevention.

## 2. Materials and Methods

### 2.1. Literature Search

This systematic review examined the use of foot orthoses in people with diabetesrelated foot disease, focusing on ulcer development and the modification of plantar pressure, given its role as a key risk factor. Following the Preferred Reporting Items for Systematic Review and Meta-Analysis PRISMA guidelines [24]. The PRISMA 2020 checklist has been included in the Supplementary Materials (Table S1). The review was not registered and any protocol was not prepared. Two research questions were formulated: Are foot orthoses effective in preventing first ulcers or re-ulceration in patients with diabetes-related foot disease? Are foot orthoses effective in reducing plantar pressure and thereby preventing ulcer onset in patients with diabetes-related foot disease? These questions were structured using the PICO format (population, intervention, control and outcomes), as presented in Table 1.

To conduct the review, the PubMed, Embase and Web of Science databases were searched using the terms “foot insoles,” “plantar insoles,” “insol*,” “foot orthoses,” “plantar orthoses,” “orthos*,” “diabetic foot,” “diabet*,” “ulcer” and “wound.” Boolean operators “AND” and “OR” were applied, as outlined in the search strategy shown in Table 2. RefWorks (EndNote fusion) was used as the reference management tool.

### 2.2. Inclusion and Exclusion Criteria

Articles in Spanish or English were included regardless of publication year. Eligible studies compared two groups of patients with diabetes and neuropathy without ulcerative lesions at baseline, or assessed the same group under different conditions, provided that at least one condition involved orthotics and another did not, independent of footwear type. Studies comparing different models of custom-made shoe insoles or inserts were also included.

The selected studies had to assess pressure reduction immediately after application of the foot orthosis, as well as prospectively in the control group. The occurrence of ulceration was only assessed prospectively.

### 2.3. Data Abstraction

Two reviewers independently extracted data, summarised in Table 3 (author and year, study type, aim, sample, intervention, study variables and results).

## 3. Results

### 3.1. Study Selection

**Figure 1:**
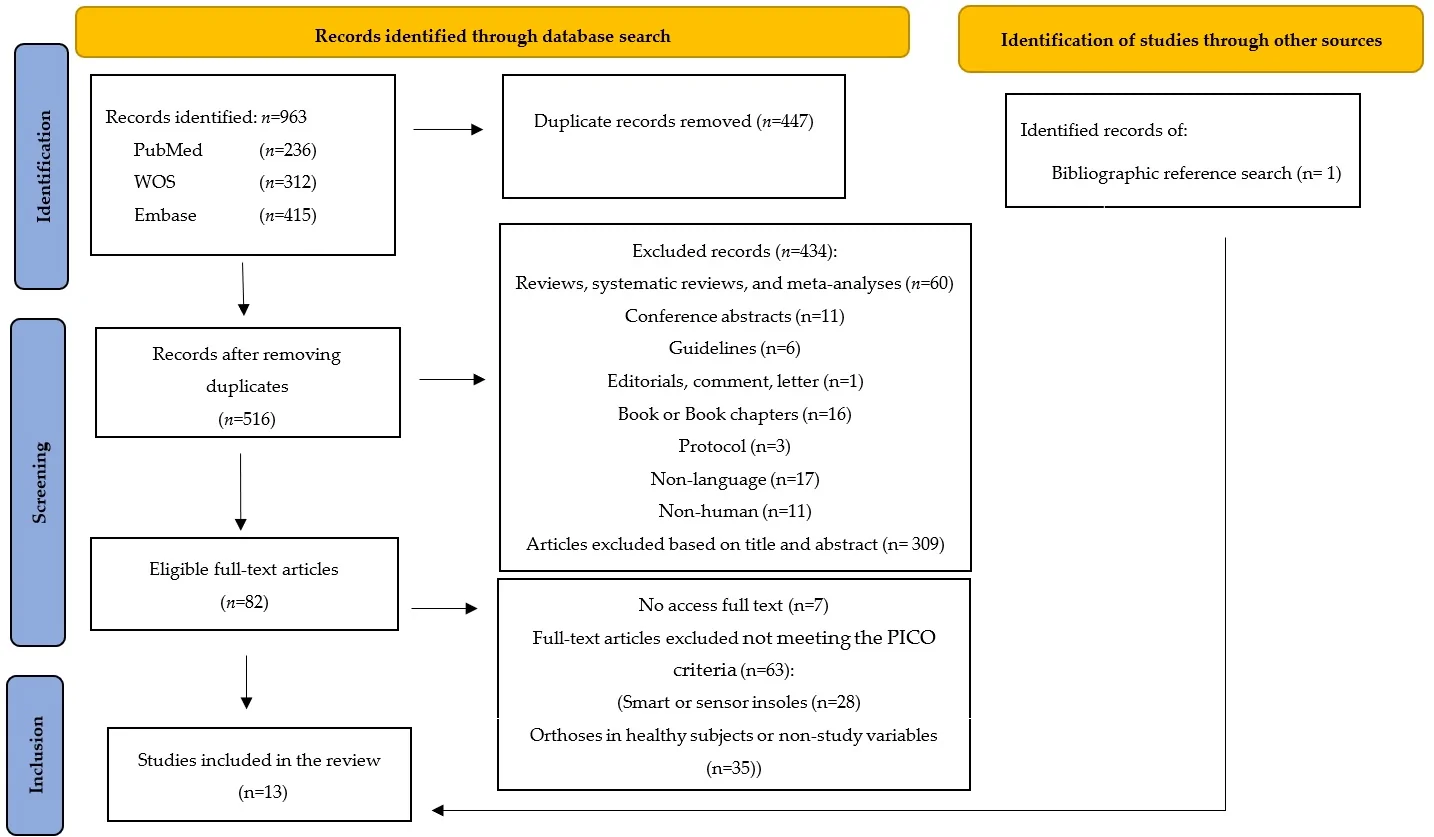
PRISMA flow diagram of the literature search and study selection.

The database search yielded 963 articles. After removing duplicates (*n* = 447), 516 references were screened. Eleven were excluded for involving animal experimentation, 17 for being written in languages outside the review criteria and 97 for not meeting the required article format (11 conference abstracts, 60 reviews or meta-analyses, 6 guidelines, 1 editorial, 16 book chapters and 3 protocols). Of the remaining 391 articles, title and abstract screening excluded 309, leaving 82 full-text articles for assessment. Of these 82 papers, 7 were excluded because the full text was unavailable and 63 were rejected because they did not meet the PICO criteria. One additional article, identified through reference search, was included. A total of 13 studies were therefore incorporated into this review, as summarised in Figure 1 and Table 3.

### 3.2. Study Characteristics

Four studies were >20 years old [27,29,32,34], three were 10–20 years old [25,31,35] and four were <10 years old [26,28,30,33]. Only three studies were published within the last 5 years [33,36,37]. All studies analysed variables related to plantar pressure [25,26,27,28,29,30,31,32,33,34,35,36,37]. Among the thirteen included studies, only two were prospective [29,32], and only Viswanathan et al. [29] assessed ulceration or re-ulceration. Regarding the orthotic devices evaluated, nine studies used customised foot orthoses [25,28,30,31,32,34,35,36,37], whereas four used insoles or non-customised orthoses [26,27,29,33]. Most studies specified the materials used in orthosis manufacture [25,28,29,30,31,32,34,35,36,37], and two evaluated stiffness or cushioning properties [26,32,36,37]. Ten studies incorporated custom-made or therapeutic footwear [25,26,27,28,29,31,32,33,34,35], which provided additional depth to accommodate orthoses appropriately.

### 3.3. Methodological Quality Assessment

To critically assess the quality of the articles, Joanna Briggs Institute (JBI critical appraisal tool) questionnaires were used. These structured questionnaires assess the internal and external validity of studies to determine whether their findings are reliable, focusing on the methodology, data collection, analysis and bias control [38]. Different questionnaires exist according to study design; for this review, which included repeated measures studies, randomised or otherwise, the appraisal tools for randomised clinical trials [39] and quasi-experimental studies [40] were applied.

Quality was determined by the number of affirmative responses in the appraisal, classifying studies as low quality or at high risk of bias (less than 50% affirmative responses), moderate quality or at moderate risk of bias (50–70% affirmative responses) and high quality or at low risk of bias (>70% affirmative responses) [41].

None of the studies selected for review were excluded based on this assessment. Most received five to seven favourable responses (55–77%), indicating moderate quality [25,26,27,29,31,32,33,34,35], whereas four studies exceeded 70% and were classified as high quality [28,30,36,37].

## 4. Discussion

The objective of this review was to assess the effectiveness of foot orthoses in modifying plantar pressure and preventing first or recurrent ulceration. Most included studies focused on changes in plantar pressure and related variables following the use of foot orthoses [25,27,28,30,31,32,34,35,36,37], while only one conducted long-term follow-up to evaluate ulceration or re-ulceration [29].

### 4.1. Plantar Pressure Modification

Overall, the use of foot orthoses, either alone or in combination with therapeutic or custom-made footwear, has been shown to reduce plantar pressures [25,27,28,30,31,32,34,35,36,37]. Arts et al. [25] demonstrated that feet with deformities showed higher plantar pressures in both barefoot and shod conditions, particularly in patients with claw toes or prominent metatarsal heads. However, these feet achieved greater percentage reductions in pressure (50–76%) when wearing therapeutic footwear and custom foot orthoses were used, with even larger reductions in areas with prior ulceration (85%, 539 kPa). Raspovic et al. [27] mirrored these findings, although their participant values varied widely (6–93%).

With longer follow-up (1 year), Lobmann et al. [32] observed that plantar pressure decreased after 2 weeks of orthosis use and remained lower at 1 year, although only the 2-week and 6-month reductions were statistically significant (32% and 28%, respectively, *p* ≤ 0.001). Despite the absence of statistical significance at 1 year, pressure was still reduced compared with the baseline (380.8 ± 190 kPa vs. 474.7 ± 183 kPa). The diminishing effect over time may reflect material fatigue, supporting the need for regular review of orthotic devices in high-risk patients.

One of the clearest areas of pressure reduction is the metatarsal heads [25,28,30,31,34,35,36,37], which bear substantial load during walking and have limited contact surface at propulsion. Muller et al. [31] attributed this immediate reduction to an approximate 30% increase in contact area produced by the orthosis, resulting in a 16–24% pressure decrease. Ashry et al. [34] further noted that this reduction was effective in both non-amputated feet and feet with first-toe amputation. Dynamic studies also demonstrated instant reductions in plantar pressure during ramp and stair negotiation [30], tasks performed routinely by many patients.

Another commonly reported variable, the pressure–time integral, also decreased with orthosis use [27,31]. This immediately noticeable reduction may help protect feet at high risk of ulceration, although it remains unclear whether the improvement is primarily due to decreased pressure magnitude or reduced load duration [27].

### 4.2. Appearance of the First Ulcer or Re-Ulceration Prevention

Although many studies reported that foot orthoses reduce pressure in areas that are at high risk of injury [27,28,30,31,35] and may therefore prevent first ulcer development or reduce re-ulceration risk, only Viswanathan et al. [29] directly assessed ulcer onset or absence after 9 months of device use [29]. Their study compared four groups of participants with diabetes: three used therapeutic sandals with different foot orthoses, and one group used their own footwear. Those without orthotic intervention demonstrated a substantially higher proportion of lesions than those with orthoses (33% vs. 4% and 3%, respectively). These findings suggest that appropriate foot orthoses reduce plantar pressure at the metatarsal heads over time and lower ulceration risk and that failure to use them leads to ineffective load distribution and increased tissue tension, which may hinder healing when injury is present.

### 4.3. Orthosis Types Used

Many of the articles in this study used custom-made foot orthoses [25,28,30,31,32,34,35,36,37], commonly manufactured from phenolic foam moulds [28,30,31] or 3D scan modelling [36,37]. Although the type of orthosis was not the primary focus in most studies [25,26,28,29,30,33], several examined the role of additional components such as retrocapital bars and internal longitudinal arches in reducing plantar pressure [28,31,34,35].

The retrocapital bar is the most frequently studied element. Zwaferink et al. [28] demonstrated a statistically significant reduction in pressure (*p* < 0.001) at the first, second and third metatarsal heads when this element was incorporated. Supporting these findings, Muller et al. [31] reported reductions of 15–20% at the metatarsal heads when the retrocapital bar was applied compared with its absence. Lott et al. [35] similarly documented a statistically significant reduction of 42 kPa at the second metatarsal head (*p* < 0.001) when the retrocapital bar was used.

Although their results were not statistically significant, Ashry et al. [34] reported pressure reductions of 48.5 kPa at the first metatarsal head and 16.28 kPa across the remaining metatarsal heads when comparing the use and non-use of this element within the plantar orthosis. They concluded, however, that despite its widespread use, the component may not consistently reduce plantar pressure. Redistribution of load towards other metatarsal regions, such as the diaphysis, may partly explain why retrocapital elements decrease pressure at the heads. Variability across studies that support its effectiveness [28,31,35] and those that do not [34] is likely influenced by differences in the element’s shape, size, placement or material.

### 4.4. Materials

Although many of the selected articles describe the materials used in the foot orthoses under investigation [25,27,28,29,30,31,32,34,35,36,37], only four examined the direct relationship between material properties and plantar pressure reduction [26,33,36,37]. In Chatzistergos et al. [26]’s earlier study, fourteen participants walked with six 3D-printed polyurethane insoles that differed only in density (10–20% in 2% intervals). Selecting the optimal stiffness for each patient produced a 46% pressure reduction compared with the control condition (no material), with optimal stiffness correlating with body mass index; higher values required stiffer materials.

Years later, Chatzistergos et al. [33] reaffirmed these findings, showing that higher plantar loads require stiffer materials to achieve effective offloading, with reductions ranging from 28% to 50% depending on anatomical location. The findings of previous studies [26,33] underscores the importance of personalised material selection to maximise offloading in patients at risk of ulceration.

As demonstrated by Ning et al. [37], 3D-printed materials (utilising resin or thermoplastic polyurethane) have been shown to exhibit a greater reduction in forces when compared to conventional materials (EVA or PORON^®^ polyurethane foam).

The remaining selected articles reported the materials used in their orthoses but did not analyse pressure-related variables in relation to these materials. Commonly used base materials included cork, ethyl vinyl acetate and Plastazote [25,28,31,32,34,35], while Plastazote, leather and PPT were frequently employed as lining materials [25,28].

### 4.5. Footwear

Although not the primary focus of this review, the footwear used in the selected studies was also considered. Most studies employed therapeutic footwear for diabetic feet, designed with extra depth to accommodate the plantar orthosis [25,26,27,28,31,32,33,34]. In some cases, this footwear alone effectively reduced plantar pressure. Lott et al. [35] reported that therapeutic footwear reduced pressure at the second metatarsal head by 9 kPa (*p* < 0.001) compared with barefoot walking, although a greater reduction occurred when a plantar orthosis with a retrocapital element was added (174 kPa, *p* < 0.004). These findings are consistent with Muller et al. [31], who also observed the greatest pressure reduction in this region when footwear and orthosis were combined (*p* < 0.001). This attenuation is likely due to load redistribution towards more proximal areas of the foot, thereby reducing strain on the plantar metatarsal heads and lowering ulceration risk.

Analysing the findings of Zwafernik et al. [28], it can be observed that, although not part of their original objective, participants in the control condition without foot orthoses wore either sports shoes or non-therapeutic footwear. Lower plantar pressures were consistently recorded with the sports shoe across all metatarsal heads (sports shoe: M1 227 kPa, M2-M3 198 kPa, M4-M5 133 kPa vs non-therapeutic footwear: M1 281 kPa, M2-M3 302 kPa, M4-M5 161 kPa, *p* < 0.05). The cushioning capacity of sports footwear likely contributed to these reduced pressures, contrasting with non-therapeutic footwear, which lacks pressure-relieving features and therefore does not attenuate load effectively.

Arts et al. [25] reported that the pressure relief achieved with custom-made footwear and personalised orthotics was less effective in patients whose plantar pressure at the foot–shoe interface was <200 kPa, a threshold commonly used to indicate ulceration risk.

## 5. Limitations, Clinical Implications, and Future Lines of Research

When formulating the research question, systematic reviews published within the past decade were considered [42,43,44]. Although this is a well-studied field, the present review differs from earlier work by focusing specifically on the use or non-use of foot orthoses in the onset of first ulcers or re-ulceration, as well as their capacity to reduce plantar pressure, irrespective of whether the devices were custom-made or prefabricated. Other offloading interventions, adherence or cost-effectiveness were not examined [42,43]. In contrast to the review by Collings et al. [44], this study concentrated solely on foot orthoses and their potential modifications. While footwear and the combination of orthoses with footwear were mentioned, alterations to shoe soles aimed at reducing pressure were not analysed. As defined in the PICO question, comparisons were limited to the presence or absence of foot orthoses and did not include evaluations of orthoses or footwear against other offloading devices [42,44]. This focus applied regardless of ulceration risk in the participants [20], whether or not they had previously had ulceration or amputation, and all included studies involved patients with diabetes and neuropathy.

The main limitations of this review were the absence of longitudinal studies that assessed ulcer or pressure reduction onset in relation to the application or non-application of orthotic treatment, or following prolonged use. Assigning orthoses to the intervention group and withholding them from controls presents an ethical challenge, which partly explains the scarcity of such studies, although further research of this type would be valuable. Another limitation relates to the publication dates of the included articles, as most were >10 years old, and their methodological quality was generally moderate, with only two demonstrating a high-quality, low-risk-of-bias profile [28,30], while the remainder fell within the moderate range [25,26,27,29,31,32,33,34,35]. Considering these constraints, future research should focus on high-quality studies evaluating the effectiveness of well-designed foot orthoses in patients with diabetes-related foot disease.

The results of this review support the use of foot orthoses as part of a preventive strategy against foot ulcers in people with diabetes, particularly those at higher risk, due to the reduction in plantar pressure they provide. They should be considered as an additional component of a comprehensive care plan for diabetes-related foot disease, alongside other elements such as regular foot assessments, therapeutic footwear, patient and carer education, and management of other risk factors.

In clinical practice, orthoses should be customised according to factors such as foot morphology, deformities, plantar pressure distribution, ulcer history, footwear and patient compliance, rather than being regarded as an isolated intervention.

Professional prescription of foot orthoses for high-risk patients should be standard practice in multidisciplinary programmes for preventing complications associated with diabetic foot syndrome. More robust future research conducted through prospective randomised studies with longer follow-up periods would provide more reliable evidence regarding their efficacy in the primary prevention of ulcers, as well as subsequent recurrences. Studies focusing on treatment adherence, cost-effectiveness, and implementation in settings such as primary care and/or multidisciplinary environments would greatly contribute to demonstrating the efficacy of this treatment.

## 6. Conclusions

In general, foot orthoses demonstrate clear effectiveness in reducing plantar pressure particularly in the metatarsal region and, based on the single study that assessed clinical outcomes, in preventing ulcer onset. However, further research is required to confirm these findings. Most orthoses in the reviewed studies were fabricated from semi-weightbearing phenolic foam moulds. In many cases, retrocapital elements were incorporated, producing substantial reductions in metatarsal head pressures. Across the studies, orthoses were predominantly made from thermoformable materials such as polyethylene foam, Plastazote or cork, with 35° ShA hardness being the most frequently reported. Therapeutic footwear was the most common footwear type used, as its increased depth accommodates orthotic devices appropriately.

## Figures and Tables

**Table 1:** PICO question.

Population	Patients with diabetes and non-ulcerated neuropathy who use foot orthoses.
Intervention	Modification of plantar pressure and/or long-term ulcer development.
Control	Patients with diabetes and non-ulcerated neuropathy who do not use foot orthoses.
Outcomes	Foot orthoses will improve plantar pressure and be effective in preventing first ulcer onset or re-ulceration in patients with diabetes-related foot disease.

**Table 2:** Search strategy in the different databases.

Search Date	Database	Search Strategy	Results
26 September 2026	PubMed	(“foot insoles”[Title/Abstract] OR “plantar insoles”[Title/Abstract] OR “insol*”[Title/Abstract] OR “Foot orthoses”[Title/Abstract] OR “plantar orthoses”[Title/Abstract] OR “orthos*”[Title/Abstract]) AND (“diabetic foot”[Title/Abstract] OR “diabet*”[Title/Abstract]) AND (“ulcer”[Title/Abstract] OR “wound”[Title/Abstract])	236
26 September 2026	Web of Science	“foot insoles” (Topic) or “plantar insoles” (Topic) or “insol*” (Topic) or “foot orthoses” (Topic) or “plantar orthoses” (Topic) or “orthos*” (Topic) AND “diabetic foot” (Topic) or “diabet*” (Topic) AND “ulcer” (Topic) or “wound” (Topic)	312
26 September 2026	Embase	‘foot insoles’:ti,ab,kw OR ‘plantar insoles’:ti,ab,kw OR ‘insol*’:ti,ab,kw OR ‘foot orthoses’:ti,ab,kw OR ‘plantar orthoses’:ti,ab,kw OR ‘orthos*’:ti,ab,kw AND ‘diabetic foot’:ti,ab,kw OR ‘diabet*’:ti,ab,kw AND ‘ulcer’:ti,ab,kw OR ‘wound’:ti,ab,kw	415

**Table 3:** Description of items.

Author (Year)	Study Type	Objective	Sample	Intervention	Variables	Results
Arts et al. [25] (2012)	Quasi-experimental.	Investigate load distribution with custom footwear at sites with maximum pressures <200 kPa in patients with a history of ulcers and identify factors influencing pressure relief inside footwear.	171 participants (336 feet, 140 male/31 female) Feet classified according to the presence or absence of deformity.	Assessment: - Without footwear. - Shoes and custom insoles. - Therapeutic footwear and custom insoles.	Maximum pressure between groups with and without deformity. Reduction in maximum pressure with and without footwear for each group.	Pressure relief is often insufficient in at-risk feet with deformities. Higher relief index in feet with known high-risk locations. Lower offloading index in feet with deformities where pressure increased in the metatarsal area.
Chatzistergos et al. [26] (2020)	Non-randomised repeated measures study.	Establish a method to calculate optimal cushioning stiffness in diabetic footwear and quantify its benefit in reducing plantar pressure in at-risk feet.	15 participants (6 male and 9 female).	Use of 3D-printed insoles made of 10 mm thick thermoplastic polyurethane filament. Six insoles with densities ranging from 10 to 20% (2% increase each compared to the previous one).	Percentage reduction in plantar pressure for each insole compared to without. Comparison of the rigidity of 3D insoles with prefabricated foams. Percentage reduction in pressure and integral pressure–time in different regions of the foot. Relationship between percentage pressure reduction and insole stiffness with the optimal stiffness for each participants. Relationship between weight or body mass index and optimal stiffness for each patient.	Optimisation of cushioning according to the participant’s needs reduces pressure in footwear. 3D orthotics offer the possibility of optimising the specific rigidity of each patient to the clinical reality. Patients with a higher body mass index require stiffer materials.
Raspovic et al. [27] (2000)	Non-randomised repeated measures study.	Evaluate pressure reduction in previously ulcerated areas with custom insoles and assess foot–insole or foot–shoe contact area, load speed and pressure–time integral with and without insoles.	8 participants (male).	Same footwear, test with and without foot orthotics.	Vertical pressure peak. Load duration. Load speed. Integral pressure–time. Contact area.	Custom-made foot orthotics reduce plantar pressure. The extent of pressure reduction varies.
Zwaferink et al. [28] (2020)	Cross-sectional analytical study.	Compare custom footwear concepts regarding plantar pressure relief and patient satisfaction in high-risk ulceration patients.	24 participants.	Test with three types of custom footwear: - Custom footwear + custom insole (A). - Therapeutic footwear + custom insoles (A, B and C).	Average plantar pressure distribution in each condition. Satisfaction level assessment using a VAS scale (comfort, fit, shoe weight and appearance).	Effectiveness of a scientific, artisanal and plantar pressure data-based approach to custom footwear design. Implementation to optimise footwear for feet at risk and prevent injuries.
Viswanathan et al. [29] (2004)	Prospective study.	Determine the effectiveness of therapeutic footwear in preventing ulcers and reducing plantar pressure, and compare types of insoles used in diabetic footwear.	241 participants (158 male and 83 female). Groups: - *n* = 100 microcellular rubber and rubber insoles. - *n* = 59 polyurethane foam, microcellular rubber and EVA insoles. - *n* = 32 participants with deformities and footwear with custom-made polyurethane, cork and EVA insoles. - *n* = 50 own footwear.	Use of therapeutic footwear with different orthotics or no use of therapeutic footwear.	Peak plantar pressure on metatarsal heads. Percentage of load and contact time on day 0 and after 9 months of use of the assigned footwear.	Non-therapeutic footwear does not reduce pressure. High recurrence of ulceration rate with non-therapeutic footwear.
Nouman et al. [30] (2017)	Case–control study.	Investigate plantar pressure in four foot zones during several gait conditions (level, ramp ascent–descent and stair ascent–descent) with and without orthoses.	16 participants (9 male and 7 female).	Test with and without insoles in: - Walking on level ground for 10 m. - Walking uphill/downhill 4 m. - Ascending/descending walking 10 stairs.	Peak plantar pressure. Contact area. Integral force–time.	Plantar orthosis reduces and redistributes pressure from the toes and forefoot to the metatarsals and midfoot. Walking on an incline produces a greater increase in pressure with and without orthotics.
Mueller et al. [31] (2006)	Repeated measures study.	Determine the effect of a plantar orthosis and retrocapital bar on peak metatarsal pressure and pressure–time integral, and identify mechanisms of pressure reduction through contact area and soft tissue thickness at the second metatarsal.	20 participants (12 male and 8 female).	- Footwear only. - Footwear with orthosis. - Footwear with orthotics + retrocapital bar.	In the metatarsal area: Peak pressure + integral pressure–time during walking and during CT scan.	Combination of orthotics and retrocapital bar achieves greater reduction in plantar pressure. The orthosis reduces pressure by increasing the contact surface. The retrocapital bar compresses soft tissue proximal to the metatarsal heads, reducing pressure on them.
Lobmann et al. [32] (2001)	Case–control study.	Evaluate the effect of specially designed footwear on plantar pressure over 1 year.	81 participants.	Intervention group (*n* = 81) participants with plantar pressure > 400 kPa, plantar orthotics + footwear. Control group (*n* = 63) participants with normal pressure and conventional footwear.	Peak plantar pressure at the time of treatment application, at 2, 6 and 12 months: - Foot area. - Heel. - 2nd–3rd metatarsal head.	Early application of orthotics is effective in reducing plantar pressure. Adjustments should be made every 6 months to prevent an increase in pressure. Control group showed marked increase in plantar pressure.
Chatzistergos et al. [33] (2023).	Randomised clinical trial.	Assess prediction of optimal cushioning stiffness from demographic and anthropometric parameters related to plantar load (sex, age, body mass index, height, shoe size).	68 participants (40 male and 29 female).	Evaluation of all participants under different conditions: - Shoes only. - Application of seven custom-made insoles of different stiffness.	Maximum peak pressure. Maximum pressure. Integral pressure–time.	Association of increased plantar load with the need for stiffer cushioning materials. Optimal cushioning stiffness preceded by five demographic-anthropometric parameters.
Ashry et al. [34] (1997)	Study of repeated measurements.	Evaluate the effectiveness of orthotic modifications in reducing maximum plantar pressures in patients with first-toe amputation.	11 participants (7 male and 4 female).	Evaluation of all participants under five conditions: - Therapeutic footwear. - Therapeutic footwear + Plastazote insole. - Therapeutic footwear + Plastazote insoles with retrocapital bar. - Therapeutic footwear + Plastazote insole with internal longitudinal arch. - Therapeutic footwear + Plastazote insole with internal longitudinal arch and retrocapital bar.	Peak plantar pressure in standing position with amputation (1st metatarsal head, lesser metatarsal heads, lesser toes and heel) and in non-amputated standing position (1st toe, 1st metatarsal head, lesser metatarsal heads, lesser toes and heel) in all five conditions.	Significantly lower plantar pressures with orthoses. No significant reduction with orthoses modified with a retrocapital bar and/or internal longitudinal arch.
Lott et al. [35] (2007)	Repeated measures study.	Investigate how therapeutic footwear and orthopaedic insoles influence pressure distribution and soft tissue strain at the second metatarsal head.	20 participants (12 male and 8 female).	Evaluation of all participants in four conditions: - Barefoot. - Therapeutic footwear. - Therapeutic footwear + orthotics. - Therapeutic footwear + orthosis + retrocapital bar.	Plantar pressure on the second metatarsal head. Percentage of soft tissue strain on the plantar 2nd metatarsal head. Pressure and thickness of soft tissue on the second metatarsal head. Coefficient of correlation between tissue deformation and plantar pressure in the 2nd radius.	Therapeutic footwear and orthotics reduce pressure and tension in soft plantar tissues of the 2nd metatarsal head. Relationship between plantar pressure and tension in soft plantar tissues of the 2nd radius.
Zhang et al. [36] (2026)	Repeated measures study.	Evaluate the use of artificial intelligence in the selection of modular materials for diabetic foot orthoses, with a view to assessing the redistribution of plantar pressure.	23 subjects (11 male and 12 female).	Evaluation of all participants under four conditions: - Barefoot. - Aritficial Intelligence material-Optimised Insole (AIO). - Double-layer Poron^®^ Medical insole (PUR). - Cork-EVA insole (EVA).	Peak plantar pressure and contact area for the whole plantar surface and heel, lateral and medial midfoot, metatarsal heads and toes.	All orthoses significantly reduced plantar pressure and increased the contact area in comparison with the barefoot condition. AIO and PUR were found to be more efficacious in reducing pressure on the metatarsal heads than EVA. Furthermore, AIO was found to be more efficacious in reducing heel pressure than PUR and EVA.
Ning et al. [37] (2026)	Repeated measures study.	Investigate the fit and distribution of plantar pressure of 3D-printed insole made from auxetic materials in people with diabetes, compared with those made from traditional materials.	23 subjects (21 females and 2 males).	Assessment of all subjects under three conditions: - Without an insole. - With a standard EVA insole. - With a 3D-designed and manufactured insole.	Assessment of dynamic plantar pressure in the toes, forefoot, midfoot and hindfoot: - Without insoles. - With two types of insoles: An insole designed and manufactured using 3D technology. A standard EVA insole for diabetics.	Using the two prototype insoles reduced plantar pressure compared to not using orthoses.

## Data Availability

No new data were created or analyzed in this study. Data sharing is not applicable to this article.

## Supplementary Materials

The following supporting information can be downloaded at https://www.mdpi.com/article/10.3390/jcm15218740/s1. Table S1: PRISMA_2020_checklist.
